# Individual Quality Explains Variation in Reproductive Success Better than Territory Quality in a Long-Lived Territorial Raptor

**DOI:** 10.1371/journal.pone.0090254

**Published:** 2014-03-05

**Authors:** Jabi Zabala, Iñigo Zuberogoitia

**Affiliations:** 1 Arrigorriaga, Biscay, Spain; 2 Icarus Estudios Medioambientales, Logroño, Spain; 3 Ornithology Department, Sociedad de Ciencias Aranzadi, Donostia/San Sebastian, Spain; University of Lleida, Spain

## Abstract

Evolution by natural selection depends on the relationship between individual traits and fitness. Variation in individual fitness can result from habitat (territory) quality and individual variation. Individual quality and specialization can have a deep impact on fitness, yet in most studies on territorial species the quality of territory and individuals are confused. We aimed to determine if variation in breeding success is better explained by territories, individual quality or a combination of both. We analysed the number of fledglings and the breeding quality index (the difference between the number of fledglings of an individual/breeding pair and the average number of fledglings of the monitored territories in the same year) as part of a long term (16 years) peregrine falcon (*Falco peregrinus*) monitoring program with identification of individuals. Using individual and territory identities as correlates of quality, we built Generalised Linear Models with Mixed effects, in which random factors depicted different hypotheses for sources of variation (territory/individual quality) in the reproductive success of unique breeding pairs, males and females, and assessed their performance. Most evidence supported the hypothesis that variation in breeding success is explained by individual identity, particularly male identity, rather than territory. There is also some evidence for inter year variations in the breeding success of females and a territory effect in the case of males. We argue that, in territorial species, individual quality is a major source of variation in breeding success, often masked by territory. Future ecological and conservation studies on habitat use should consider and include the effect of individuals, in order to avoid misleading results.

## Introduction

That individuals differ in their ability to survive and reproduce is a central issue of selection theory. Evolution by natural selection depends on the relationship between individual traits and individual fitness [1]. Although studies of vertebrates reported that most variation in individual fitness is accounted for by the variation in offspring survival and in lifespan [2], [3], rather than by variation in fecundity, fitness is often equated with a tendency to leave more or fewer offspring [1], [4].

One aspect of animal ecology often studied to assess its effect on fitness and reproductive success, is habitat selection [4]. Habitat preferences are assumed to be adaptative, so fitness is higher in preferred habitats [5], and local habitat affects the fitness of individuals through variation in resources and environmental conditions [6]. Although fitness is an individual measure, several authors combined the concepts of habitat and fitness into the notion that habitat confers fitness to its occupants, and this has been regarded as the habitat fitness potential [7]. This concept is well supported by studies reporting differences in reproductive success in birds and other groups using different environments [4], [8], [9]. In the case of territorial species, territory quality may affect individual fitness [10]. Accordingly, individuals may benefit by recognizing spatial variation in habitat quality and settling accordingly and it is widely assumed that both clutch size and reproductive output in territorial birds depend on the quality of their territories [4], [10], [11]. In fact, habitat heterogeneity and despotic settlement have been postulated as the main mechanism of population regulation for territorial birds [10], [12], [13], [14]. Under an ideal free distribution equal competitors select habitats to maximize their individual fitness, whereas under an ideal despotic distribution or more complex distribution models, individuals are unequal competitors and the resources or territories of highest quality are monopolized by the strongest competitors [4]. Several studies report higher reproductive success in preferred habitat territories [5], [10], [15], and long-lived raptors have been reported to move from low–quality to high–quality areas as they age [15]. However, this rule is not always followed and other factors, such as individual quality, are invoked in order to explain results (e.g. [16], [17]).

One criticism that can be raised with regard to most studies on habitat-territory quality is that they treat conspecific individuals as ecologically equivalent, estimating fitness for groups [1], [18], regardless of the fact that individuals of the same species may use different resources. Habitat quality can depend on the resources used and the skills possessed by individuals, thus confounding habitat and individual quality. Inter-individual variation has been ignored in many ecological studies, in spite of individual specialization being widespread, and capable of profoundly affecting population ecological and evolutionary dynamics [18]. Additionally, several studies have reported important intra group differences and instances of variation in fitness which are attributable to individual traits [19], [20]; see [18] for a review. In the case of raptors, some authors suggest that, although site quality is a major determinant of fitness, its effects can be confounded with individual quality, a relationship that has rarely been studied in large, long-lived vertebrates [20], [21]. However, individual quality can vary with age [22], [23], and productivity can also be influenced by trophic breadth [24]; [25] and other denso-dependent and denso-independent factors [26]

In this study we aimed to analyse the interaction and contribution of territory and individual quality to the fitness of long-lived, territorial raptors. For this purpose we used a long term monitoring data set of peregrine falcons and compared three competitive hypotheses to explain variation in reproductive success: (1) variation in reproductive success results from differences in territory quality; (2) variation in reproductive success results from differences in individual quality; and (3) variation in reproductive success results from the combined effects of territory and individual quality.

## Materials and Methods

### Study area

The study area covered the area of Biscay (Basque Country, northern Spain; 2,384 km^2^; 43°12’00’’N 3°13’00’’W). The territory is hilly and steep, with only 50 km separating the sea from the highest altitude (1,480 m a.s.l.). Man-made forests, pastures, small villages and densely populated cities make up the bulk of the province More than 50% of the area is dedicated to forestry, mainly plantations of *Pinus radiata* and *Eucalyptus* spp.s spreading at the expense of traditional small-scale farming. The climate is temperate, with an annual rainfall of 1,000–1,300 mm. and mean annual temperatures of 11–12°C.

### Field procedure

The peregrine falcon population in Biscay was systematically surveyed each year from 1997 to 2012. The monitoring focused on 37 pairs, although the population fluctuated between 42 and 52 breeding territories [27]. Falcons were first located 30 days before the earliest local laying date recorded (20^th^ February, [28]). They can be readily observed at this time since they engage in frequent courtship displays. Nests were located by observing displaying individuals near the crags and sea cliffs where they eventually breed. We defined territories as being the exclusive nesting sites plus the surrounding areas and resources defended by territory holders against intruders. We did not define territories as surface areas of a given size because the radio-tracking data from different individuals showed marked variation in the home range used [28]. Additionally, no two pairs ever nested in the same area and nests belonging to different territories sites were separated by 4.25 km on average [28].

Three different methods were employed to identify adults on an individual basis: (1) between 1997 and 2013, 636 fledglings and 30 either subadult or adult birds were ringed with official alphanumeric coloured rings. Of these, 30 males and 15 females were recruited into the breeding population [29]. (2) Individual photographs (Canon 7D camera with 50–500 mm lens, or digiscope arrangement with a Swarovski 20×60 ATS80 telescope) of 33 breeding males and 32 females were taken, in order to identify non-ringed peregrines using their individual markings. (3) Drawings of details were made and the colour of the face, moustache and breast, plus any behavioural aspects, were noted for every territorial peregrine (see [9], [27], and for a detailed description of the method [29]). Overall, 83 breeding males and 96 females were identified, and the identity of monitored individuals was checked against colour ring records, photographs and drawings each year. The reliability of identifications made from photographs and drawings was checked against that obtained from the coloured rings. For this purpose, in territories occupied by ringed individuals (17 males and 9 females), we first identified breeders from their individual features and from drawings and photographs. These identifications were later checked against the ring readings. In some instances during the study period, identification using drawings suggested changes in territory holders which could then be ascertained by checking the rings the of new breeders. The Agriculture and Environment Departments of the Diputación Foral de Bizkaia issued the licences to work with the species, to capture, handle and ring individuals, and to conduct fieldwork across the whole study area.

### Variables considered in the models

To control for additional factors that could affect reproduction, other than territory and individual identity, a set of weather and nesting site variables and the estimated experience of the individual were considered for each breeding attempt. Some pairs used alternative nests within their breeding territory during the study period. In these instances the variables of the nests used each year were measured but the same territory identity was retained as a random factor.

April rainfall has an important effect on the reproductive success of peregrines in the area [30]. Therefore, since there are 19 meteorological stations available within the study zone, the data from the one closest to each nest was used when including April rainfall as a predictor variable. To include the effect of shelter from the weather, the degree of cover offered by the nest was described on a 1 to 3 scale: (1) exposed nests without shelter, built on ledges or in open crevices in cliffs; (2) nests with overhanging rocks or situated in small crevices that offered better protection; (3) the best sheltered nests (deep crevices in rocks and small caves). The height of the nest on the cliff was also considered as a possible predictor of disturbance and/or predation by small carnivores or others.

Previous studies found a disadvantageous effect of competition for the nesting site with other large raptors [31]. Of the potential competitors, only eagle owls (*Bubo bubo*) were present in the study area, although at very low densities (from 3 to 10 breeding pairs). Therefore, we did not consider other raptors as a possible cause of differences in reproductive success. Since territory quality could not be ascertained independently from individual quality [32], we used territory identity as a surrogate. We assumed that if territory quality is a determinant of reproductive success, good territories would enable greater breeding success for any territory holder and territory identity would accommodate variation in breeding success. On the other hand, under the hypothesis of individual quality being the main driver of variation in breeding success, good quality individuals would have high reproductive success independently of territory. Therefore, we used individual identity as a surrogate of individual quality. An effect of experience on breeding success, and sometimes on subsequent survival, has been found in several species (review in [33]). To model a possible learning effect of experience within individual variation we included the number of years for which a given individual was known to be occupying a territory. For individuals that entered into the breeding population during the study, its value was clear. However, at the onset of the monitoring there were several individuals already reproducing whose experience as breeders could not be ascertained. Those individuals were assumed to be in their first reproductive season if when the monitoring started they were aged as second or third calendar year (using plumage characteristics described in [28 and 34]). Individuals that were aged as adults and were already breeding in the first monitoring year, were assumed to have some breeding experience and considered to be making their second breeding attempt. The first reproductive attempt might result in selection against low quality first time breeders, with associated lower success and higher adult mortality [35]. To avoid confusion due to selection against poor quality individuals in the first year, and to avoid fitting individual variance groups for single data, individuals that were monitored only once were not considered. In the case of breeding pairs, experience was included as the mean of the breeding experiences of the male and the female.

### Model specifications and response variables:

The data were analysed with Generalised Linear Mixed Models (GLMMs), in which fixed factors quantified the effects of environmental variables and random effects quantified variation across territories and individual falcons, while accounting for pseudo correlation [36], [37]. GLMMs have been used extensively to test the significance of fixed factors, with random effects being regarded as a nuisance. However, the latter may explain variance and heterogeneity, and hence are often of interest to ecologists. GLMMs also offer an opportunity to evaluate and assess hypotheses involving random effects [37].

A fixed effect structure was built using the above-mentioned variables and was used in every model. Three possibilities for variation in reproductive success were considered: (1) Variation in reproductive success is best explained by territory quality. In this scenario high quality territories support high reproductive success, regardless of breeder quality. This hypothesis was modelled by adding territory identity to the structure as a random factor. (2) Variation in reproductive success is best explained by individual quality. This hypothesis was modelled by adding individual identity as a random factor. In this case, breeders would use the resources of the territory in different ways, or with different efficiency levels, and variance in reproduction would be better explained by breeders rather than by territory. (3) Reproductive success is the result of the interaction between territory and individual quality: high quality territories support higher breeding success, while low quality territories support lower reproductive success, with additional variation being attributable to individual quality in both situations. This third hypothesis was modelled with a random structure in which individual identity was nested within territory identity. It incorporates both previous models and gives information about the significance and direction of effects of both random factors. It also considers whether the variance explained by each of the variables is additive or compensatory. These three hypotheses are not mutually exclusive, but we compared them under an information-theoretic approach, to elucidate which was best supported by the data and highlight any possible interactions between territory and individual and their relative strengths [32], [37], [38].

Two different response variables were used: (1) the number of fledglings produced each year, and (2) a Breeding Quality Index (BQI) defined as an individual’s ability to produce offspring compared to the average success of others in the same year. BQI was calculated as the difference between the number of offspring of a particular individual/territory and the average number of offspring of the monitored territories in the same season. In 2006, for instance, the monitored pairs produced an average of 2.2 fledglings per pair; therefore the BQI values of pairs that produced 2, 3 and 4 fledglings were –0.2, 0.8, and 1.8 respectively. To assess the importance of individual identity three approaches were used: (1) breeding pair identity, in which pairs consisted of unique combinations of male and female identities; (2) male identity, with random effects grouped after the identity of the male in the breeding pair; and (3) female identity, with the identity of the female in the breeding pair as the random effect. Therefore, six sets of models were analysed: three using the number of offspring as the response variable, with the random factor effect defined as per the three approaches above, and another three using BQI as the dependent variable and including the different random factors for individual effect.

In order to minimise the possible effect of other variables on reproductive success we evaluated the convenience of models including other sources of correlation. Temporal effects were an important source of possible correlation in the data. These were not of interest in our case, but could cause longitudinal correlation (with the reproductive success of one year being correlated to that of the previous year, either positively because of abundance of prey items being correlated from year to year, or negatively, with high investment in offspring in one year being related to lower investment the following year), or a transversal effect, with good years of high reproductive success for all breeding pairs, and bad years with poorer outcomes in all cases [26]. To control for this, another competitive model with “year” as a random factor was included in every set of models, as well as models in which the territory and/or individual were nested within year.

### Data analyses

The reliability of peregrine identification using photographs and drawings was tested against colour ring readings using the χ^2^ test with the Yates correction for contingency tables [39] . Serial correlation in reproductive success over different years was evaluated by analysing autocorrelation and partial autocorrelation on the average fledglings produced each year, using the “acf” function implemented in R [40], .

Correlation between the average productivity of individuals and territories and their coefficient of variation was analysed in order to search for evidence of good individuals and territories [10]. When evaluating the models the convenience of error structures was firstly assessed using Generalised Least Square (GLS) models without error structures, comparing their fit against GLMMs built with package “nmle” using a Gaussian family and an Identity link [36], [42]. Models were compared based on Akaike Information Criterion corrected for small sample size (AIC*c*) (see below), to determine if the inclusion of random effects was supported by evidence. Six possible random effect structures were evaluated: (1) individuals, (2) territory, (3) individuals nested within territories, (4) individuals nested within year, (5) territories nested within year, and (6) year. This comparison was performed for the three different specifications of the individuals concerned: breeding pair identity, female identity, and male identity. We used these three specifications to gain insight into possible sex-related differences and to get the best information from our data, which in some cases did not include information on the identity of both members of the breeding pair, with only the identity of one bird being known.

Following this, in models using the number of fledglings successfully raised as the response variable, a Poisson model with a log link was employed, in order to ascertain which factor was better at explaining variability in reproductive success. The number of data per group of random factors was often lower than 5, therefore the Laplace approximation [37] was implemented using the R package “lme4” [43]. In models using BQI as the response variable, different AIC*c* values were compared. Once the best model in each set had been determined it was checked for overdispersion, normality of the residuals was evaluated using the Shapiro-Wilks test, and graphical checks of their distribution against fixed factors and fitted values were performed [36], [40].

### Model selection and multi-model inference:

To select the model best supported by our data Akaike’s Information Criterion, corrected for small sample size (AIC_c_), was used. We calculated AIC_c_ differences between candidate models and selected the model showing the lowest AIC_c_ value as the best one_._ Models within 2 units of ΔAIC_c_ were considered to have substantial empirical support, models with ΔAIC_c_ 4 – 7 were considered to have substantially less empirical support, whereas models with more than 10 units of ΔAIC_c_ were considered as not supported by the data. We also calculated the Akaike Weights *w_i_* (i.e. the weight of evidence in favour of that model being the best in the set) for the candidate models [38].

## Results

### Peregrine falcon individual identification and monitoring

We recorded 415 reproduction attempts in which the identity of the individual was known. In 77 cases (47 male and 30 female) individual identity was firstly determined using photographs or drawings and later confirmed by colour ring readings. Similarly in 43 cases, after making photograph and drawing comparisons one breeding animal was determined to have been substituted (9 occasions where a non- ringed animal was substituted by a ringed one; 12 instances of ringed animals substituted by other ringed animals; and 19 cases of ringed animals substituted by non-ringed ones), and the change was later verified by the presence or lack of colour rings. All of the identifications from photographs and drawings that could be checked by rings, proved to be accurate. The performance of this identification system was statistically significant (χ^2^_yates_ = 116.355; d.f. = 1; p<0.001). For 363 breeding attempts (199 female and 164 male attempts), involving 125 different individuals, we were able to estimate the experience of the individual. We recorded 223 reproductive attempts for which the identity of at least one individual of the reproductive pair was known, and 140 occasions on which we knew the identity of both birds. During the study period, none of the individuals that entered a particular territory as a breeder ever moved to another territory.

After discarding, for the purposes of analysis, those territories and breeding pairs for which we had complete information for only a single occasion (occasional breeding territories or those which were not intensively monitored), the resulting main data set consisted of 110 reproductive attempts with complete information. These attempts involved 31 different pairs from 18 separate territories. The monitoring results for pairs and individuals are summarised in Table 1 (see also Table S1 for further detail).

**Table 1:** Monitoring summary, considering only cases with complete information and two or more consecutive years of monitoring.

	Breeding pairs		Females		Males	
	Territories	Pairs	Territories	Individuals	Territories	Individuals
*n*	18	31	27	38	22	37
Average	6.11	3.55	6.18	4.39	6.59	3.92
St. Dev	3.50	2.05	3.39	2.80	3.90	2.11

Breeding pairs refers to the couples in which the identity of both members was known, whereas Females and Males refer to data sets considering only one member of the breeding pair. Territories represents the different territories included in the analyses and Pairs/Individuals the breeding pairs or individuals. *n* indicates the number of territories/pairs/individuals included in the analyses. Average is the average number of years for which each territory/pair/individual was successfully monitored and St. Dev. its standard deviation.

### Reproductive success, correlation and convenience of random factors

**Figure 1:**
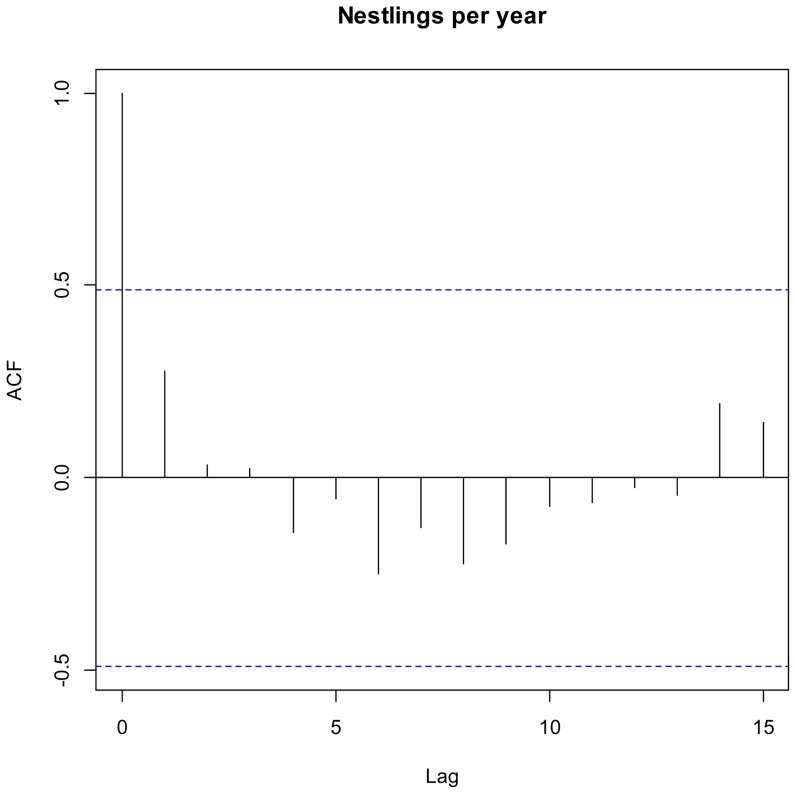
Serial correlation plot of average fledglings produced per year. Lag is expressed in years.

**Figure 2:**
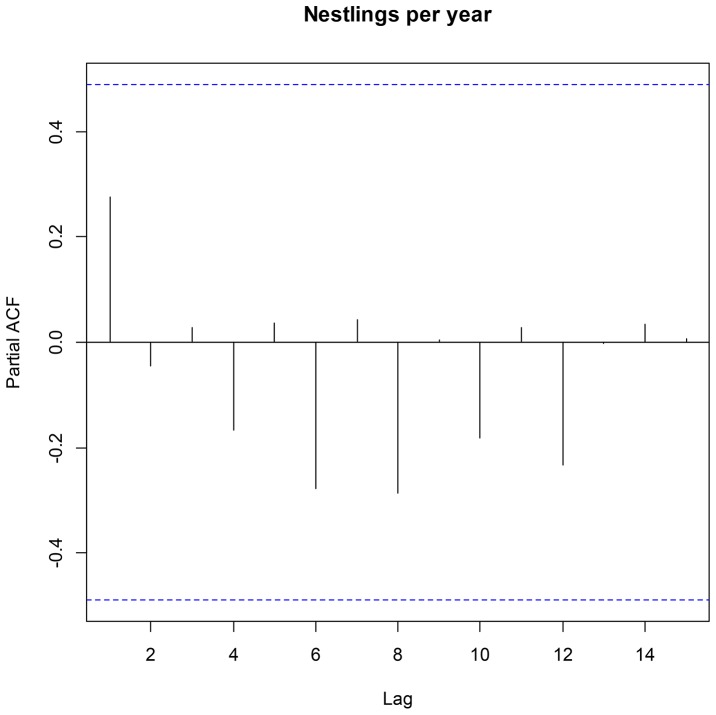
Serial partial autocorrelation correlation plot of average fledglings produced per year. Lag is expressed in years.

The number of fledglings produced by different territory holders, and also their BQI varied markedly between and within territories (details in supplementary material). For instance, in te territory “number 2” one breeding pair, monitored for 3 years, had a positive BQI of 0.95 whilst other pairs that occupied the territory had negative BQI values. This said, there was a negative correlation between the average productivity in a territory and its coefficient of variance (CV = 1.91–0.568*average productivity; R^2^ = 0.815; p<0.01; n = 18), as well as between the average productivity of a breeding pair and it is coefficient of variance (CV = 1.84–0.5138*average productivity; R^2^ = 0.675; p<0.01; n = 28 - three breeding pairs produced no fledglings during the monitoring period and therefore their CV could not be calculated), suggesting good reproduction in good territories and good reproduction by good breeders. This pattern held when using the data from unique breeding pairs, males only, and females only. The analysis of serial temporal correlation showed no clear pattern or correlation structures in the reproductive success data (Figure 1), and the same result was obtained for the partial autocorrelation (Figure 2). We therefore discarded Auto Regressive Moving Average models and serial correlation structures from the analyses [36], [40].

### Model comparisons

When we compared models using number of fledglings as the response variable and different error structures representing the different hypotheses against a GLS model, we found, in all cases strong evidence in favour of the models including random terms. In most instances the AIC*c* values supported models including individual identity (either the breeding pair identity, male identity or female identity) as a random term, while models with no random terms or with other random structures had scarce or virtually no support from the data (Table 2). In the Female data set, there was also strong evidence in favour of the model considering year as a random term, this model being the best model in the set. Models whose random terms included breeding pair identity or individual identity nested in other factors, as well as models with year as a random term in the Female data set, were within some 2.4 AIC*c* units of the best model (Table 2). Nonetheless, these model specifications were similar to the best one, although they included one more parameter (the nested random factor) and hence were not considered further [38]. The considerable increase in AIC*c* of the models that included year as a random factor did not support a transversal effect of year on reproductive success (i.e good years and bad years) for the Breeding pair and Male data sets, but this was supported in the case of the Female set (Table 2).

**Table 2:** Models with different random structures compared with a GLS Model.

Data set	Random term	AIC	AIC*c*	*n*	K	ΔAIC*c*	*w_i_*
Breeding pairs	GLS	414.333	415.431	110	7	6.149	0.020
**Breeding pairs**	**Male identity**	**407.846**	**409.282**	**110**	**8**	**0**	**0.425**
Breeding pairs	Breeding Pair identity	408.665	410.091	110	8	0.809	0.283
Breeding pairs	Male ID + Female ID	409.846	411.646	110	9	2.364	0.130
Breeding pairs	Female identity	413.22	414.656	110	8	5.374	0.029
Breeding pairs	Territory identity	416.221	417.646	110	8	8.364	0.006
Breeding pairs	Breeding Pair ID nested in Territory ID	410.665	412.465	110	9	3.183	0.086
Breeding pairs	Breeding Pair ID nested in year	417.043	418.469	110	8	9.187	0.004
Breeding pairs	Territory nested ID in year	417.043	418.469	110	8	9.187	0.004
Breeding pairs	**Year**	**415.043**	**416.469**	**110**	**8**	**7.187**	**0.012**
		Female					
Female	GLS	610.236	610.940	167	7	7.517	0.012
Female	Female identity	606.017	606.929	167	8	3.505	0.090
Female	Territory identity	611.163	612.074	167	8	8.650	0.007
Female	Female ID nested in Territory ID	608.017	609.164	167	9	5.740	0.030
Female	Female ID nested in year	604.512	605.659	167	9	2.235	0.170
Female	Territory ID nested in year	604.512	605.659	167	9	2.235	0.170
Female	Year	602.512	603.424	167	8	0	0.521
		Male					
Male	GLS	545.514	546.332	145	7	5.378	0.045
Male	**Male identity**	**539.895**	**540.953**	**145**	**8**	**0**	**0.669**
Male	Territory identity	546.390	547.449	145	8	6.495	0.026
Male	Male ID nested in Territory ID	541.895	543.228	145	9	2.275	0.215
Male	Male ID nested in year	548.313	549.646	145	9	8.693	0.009
Male	Territory ID nested in year	548.313	549.646	145	9	8.693	0.009
Male	Year	546.313	547.371	145	8	6.418	0.027

The response variable used was number of fledglings. Data set indicates whether the data refer to the breeding success of pairs, females or males. Random term indicates the random structure depicting the model (all models had the same fixed term structure). AIC is the Akaike Information Criterion and AIC*c* is the same corrected for small sample sizes. *n* stands for the number of data included and K for the number of parameters of the model. ΔAIC*c* is the increase in AICc units respective to the best model, and *w_i_* the Akaike weight, or the weight of evidence in favour of that model being the best model in the set. The best model in every set is marked in bold.

After finding empirical support for the inclusion of random terms, models with competitive random structures were evaluated using the R package lme4 [43] (Table 3). Considering these, we found that models including individual identity as a random factor were the best models in the Breeding pairs and Male data sets. In the Female data set the best model was that including the year as the random term (Table 3). Notwithstanding, in that data set the model which included individual identity outperformed the territory model. For the Breeding pair and Male data sets, the empirical support for the best model was substantial, particularly in the Male set (Table 3). Graphic validation of the best models obtained with “lme4” showed lack of normality in the residuals in every case (Breeding pairs: W  =  0.9616, p  =  0.003; Females: W  =  0.9492, p < 0.001; Males W  =  0.9511, p < 0.001), and patterns in the residuals were observable when plotted against fitted values, indicating heterogeneity [36]. This suggested that there were sources of correlation which were not controlled for in the data, and that some important explanatory variables might be missing.

**Table 3:** Results of multi-model evaluation of different random structures applied to number of fledglings and fitted using the Laplace approximation.

Random term	AIC	AIC*c*	*n*	K	ΔAIC*c*	*w_i_*	Overdisp. Factor (*c*)	*p* overdisp
**Male identity**	**157.965**	**159.063**	**110**	**7**	**0**	**0.320**	**0.831**	**0.892**
Breeding Pair identity	158.256	159.354	110	7	0.291	0.550	0.835	0.886
Male ID + Female ID	159.965	161.390	110	8	2.327	0.100	0.839	0.878
Breeding Pair ID nested in Territory ID	160.256	161.681	110	8	2.328	0.172	0.844	0.871
Female identity	160.808	161.906	110	7	2.843	0.080	0.942	0.647
Territory identity	161.020	162.118	110	7	2.764	0.138	1.037	0.379
Year	160.991	162.089	110	7	2.735	0.140	1.011	0.451
Female identity	234.899	235.603	167	7	3.377	0.212	0.834	0.991
Territory identity	236.281	236.985	167	7	4.760	0.098	0.985	0.820
Female ID nested in Territory ID	236.899	237.810	167	8	5.378	0.078	0.839	0.989
**Year**	**231.521**	**232.226**	**167**	**7**	**0**	**0.612**	**0.870**	**0.931**
**Male identity**	**228.955**	**229.773**	**145**	**7**	**0**	**0.676**	**0.941**	**0.948**
Territory identity	234.873	235.691	145	7	5.918	0.037	1.144	0.180
Maleil ID nested in Territory ID	230.955	232.014	145	8	2.000	0.249	0.948	0.941
Year	234.694	235.511	145	7	5.739	0.038	1.118	0.345

The first set of models uses breeding pairs to identify breeding units within territories, while the second and third refer to Female and Male data sets. Random term indicates the random structure depicting the model (all models had the same fixed term structure). Overdisp. Factor is the estimated overdispersion factor and *p* overdisp. shows the probability of the data being overdispersed. The best model in every set is marked in bold. For others items, the same key as in Table 2 is used.

In GLMMs using BQI as the response variable, the best model in every set was that including individuals as the random factor (Table 4). In the Breeding pairs data set the model including male identity as a random effect was the best model, having substantially more support from the data than models with other random effects. The model including breeding pair identity ranked second, but was more than 2 AIC*c* units away from the model including male identity (Table 4). The results from the Female data showed no effect of year, and two models performed within two AIC*c* units of the best one (Table 4). Finally, the Male data produced similar results: the model including male identity as a random term performed best, while models considering territory, and individual nested in territory, were still within 2 units. Other models had substantially less empirical support (Table 4). Residuals of the best model in each set only departed from normality, in statistical terms, in the case of the males (Breeding pairs: W  =  0. 9828, p  =  0.170; Females: W  =  0. 9858, p  =  0.088; Males W  =  0. 9774, p  = 0. 012). Graphic validation of the best model in each set showed that patterns observed in the residuals of the models when using number of fledglings as the response variable (see above) faded in BQI models. Some could still be intuited, suggesting residual non-modelled correlation in the data. No patterns appeared in the residuals when plotted against fixed effects.

**Table 4:** Results of multi-model evaluation of different random structures and GLS models using BQI as the response variable. Same key as in Table 2.

Data set	Random term	AIC	AIC*c*	*n*	K	ΔAIC*c*	*w_i_*
Breeding pairs	GLS	393.388	394.486	110	7	2.225	0.131
Breeding pairs	**Male identity**	**391.162**	**392.261**	**110**	**7**	**0**	**0.400**
Breeding pairs	Breeding Pair identity	392.912	394.338	110	8	2.077	0.141
Breeding pairs	Male ID.+ Female ID	393.162	394.589	110	8	2.328	0.125
Breeding pairs	Territory identity	395.177	396.603	110	8	4.342	0.046
Breeding pairs	**Female identity**	**395.388**	**396.486**	**110**	**7**	**4.225**	**0.048**
Breeding pairs	Breeding Pair ID nested in Territory ID	394.912	396.712	110	9	4.451	0.043
Breeding pairs	Breeding Pair ID nested in year	397.388	399.188	110	9	6.927	0.013
Breeding pairs	Territory ID nested in year	397.388	399.188	110	9	6.927	0.013
Breeding pairs	Year	395.388	396.814	110	8	4.553	0.041
		Female					
Female	GLS	577.657	578.361	167	7	0.497	0.238
Female	**Female identity**	**576.953**	**577.864**	**167**	**8**	**0**	**0.305**
Female	Territory identity	577.574	578.485	167	8	0.621	0.224
Female	Female ID nested in Territory ID	578.903	580.050	167	9	2.186	0.102
Female	Female ID. nested in year	581.657	582.803	167	9	4.939	0.026
Female	Territory ID nested in year	581.657	582.803	167	9	4.939	0.026
Female	Year	579.657	580.568	167	8	2.704	0.079
		Male					
Male	GLS	526.226	527.044	145	7	2.773	0.110
Male	**Male identity**	**523.212**	**524.271**	**145**	**8**	**0**	**0.416**
Male	Territory identity	524.996	526.055	145	8	1.784	0.236
Male	Male ID nested in Territory ID	524.915	526.249	145	9	1.977	0.179
Male	Male ID nested in year	530.226	531.560	145	9	7.288	0.012
Male	Territory ID nested in year	530.226	531.560	145	9	7.288	0.012
Male	Year	528.226	529.285	145	8	5.014	0.036

Estimates of the standard deviation (SD) of random factors from model outputs were inspected (Table 5). In fledgling based models fitted with the Laplace approximation, SD ranged between 0.285 and 0.344, therefore on 95% of occasions individual identity could explain differences in productivity of the order of approximately ± 0.6 fledglings. Regarding BQI based models, the SD of individual identity was, on average, some 0.35 units, accounting for approximately ± 0.68 BQI units in the 95% of the occasions. This was around 1.5 times the SD accounted for by territory, and supposed 41.7% of the residual variance (42.0, 38.3, and 44.8% for Breeding pair, Female and Male data sets respectively).

**Table 5:** Standard deviation of the random factor.

Random factor: Individual or Breeding pair Identity				
		Data set		
*Response Variable*		*Breeding Pair*	*Female*	*Male*
Fledglings	intercept	0.285	0.267	0.344
BQI	intercept	0.481	0.440	0.540
	Residual	1.145	1.149	1.206
				
Random factor: Territory				
		Data set		
*Response Variable*		*Breeding Pair*	*Female*	*Male*
Fledglings	intercept	0.000	0.000	0.145
BQI	intercept	0.248	0.375	0.415
	Residual	1.211	1.169	1.253

We show the standard deviation of the best model in each set (in all cases this was the model including either individual or breeding pair identities, see Tables 3 and 4), and that of the same model set but with a random factor specification based on territory.

We inspected fixed factors in the best model of each set. None reached statistical significance in analyses of BQI (Table 6), whereas when analysing the number of fledglings the amount of rainfall in April was significant in every instance. Furthermore, nest shelter also reached statistical significance in the Breeding pair data set.

**Table 6:** Brief results of the fixed factors in the best model in each set (see Tables 3 and 4).

Response variable		BQI			Fledglings		
Fixed Factor		Breed. Pairs	Female	Male	Breed. Pairs	Female	Male
Rain fall		0.089	0.080	0.100	**0.004**	**0.004**	**0.001**
Est. Exp.		0.117	0.247	0.085	0400	0.142	0.290
Nest height		0.792	0.454	0.905	0.219	0.880	0.631
Nest shelter:	medium	0.062	0.127	0.067	**0.045**	0.080	0.110
	high	0.070	0.061	0.065	**0.044**	0.119	0.069

In the BQI section we show the *p*-value of the t statistic, while in the Fledglings section it is the *p*-value of the z. *p* values beyond 0.05 are marked in bold.

## Discussion

Our results supported the hypothesis of an individual effect rather than a territory effect on variation in reproductive success. Including an individual identity as the random factor improved the model with no random factors in every case, whereas including territory identity did not. Models including breeding pair or male identity as the random factor were the best in their sets when using number of fledglings as the response variable, while in the case of the females the best model was that considering the year as a random effect, female identity being second. When considering BQI as the response variable, the best model was always that including individual identity as a random factor, either as male identity, female identity, or unique breeding pair identity. The latter, however, performed worse than the identity of the male of the breeding pair. Models based on the individual BQI of males and females offered some support for a territory effect, with models including territory identity as a random effect performing within two AIC*c* units of the best model, and better than the GLS model in the Male data set (Table 4). The evidence in favour of an individual effect was higher for males than for females, and in the Breeding pair data set male identity was the random factor that best accommodated variation. It also clearly outperformed female identity. A plausible explanation for this could arise from the difference in roles during the early reproductive period. During this time females spend more time on the nest and food supplies for both the female and the fledglings depends on the male’s hunting skills, with the latter regularly delivering prey to the nest [30], [44]. Therefore, males that fit poorly to the characteristics and prey of their territory may lose fledglings, while males skilful in exploiting their territory provide enough food to raise all their fledglings. However, an important degree of colinearity might exist between male identity and female identity as a consequence of mate conservatism. We could not ascertain to what extent our results were affected by this colinearity. It is clear however, that male identity accommodates most of the variation in breeding success and therefore is the most plausible cause. In the case of females, the lesser support obtained could arise from the different roles of reproduction, or be a consequence of repeated breeding occasions with the same male. However, long-term studies with passerines found an effect of female quality on fitness, considerably higher than that of habitat [32]. Indeed, in our case, in the Female data set, female identity was the factor that best accommodated variance in BQI. Therefore, we could argue that female quality and not territory quality causes variation in relative breeding success, but the issues on colinearity raised above prevent strong conclusions. Contrastingly, variance in fledglings was best explained by year, suggesting an effect of good and bad years for females. Notwithstanding, this year effect faded when considering breeding pair identity, suggesting that the hunting abilities of peregrines, especially in the case of males, can overcome the main drawbacks of poorer years, either because high quality males can provide enough food for hatchlings even under harsh conditions, or also as a result of the selection by peregrines of partners of similar quality.

There are three important factors that could affect individual quality and productivity; diet breadth, weather, and the age of the individual [18], [22], [24], [25], [30]. Several studies on raptors found a positive relationship between productivity and diet diversity [24], [25], whilst others found higher fecundity in individuals with specialised diets [18]. The peregrines breeding in our study area feed intensively on migratory birds *en route* to their breeding grounds. In adverse weather years, main migration streams might stop for several days causing peregrines to switch to other food sources and exploit different prey groups and sizes [30], probably with different hunting techniques. The capability to switch to alternative prey during periods of low migration passage, or long spells of bad weather, could explain why there was no evidence of a “year” effect for the males when fledglings were used as a response variable. In fact, field observations showed instances of birds with high breeding success specializing in different prey [30]. Previous studies found that raptor males provide females and offspring with alternative prey items when the staple prey is in short supply and that experienced males may be better than younger individuals at achieving this [45], [46], [47]. In our case, higher quality males seemed to deliver more prey in all circumstances regardless of territory. An interesting question, not answered by our data, is whether a given individual might perform well in one territory and poorly in another.

The relationship between rainy spring seasons and lower reproductive success is well established for our study population, as well as for other raptors [27], [48], [49]. Harsh winter conditions can result in poorer body conditions, less energy available for egg production and a reduction in the number of eggs laid. The relationship between hard weather conditions, lower resource availability, and a reduced clutch size and breeding success is well documented in several raptors species [46], [50], [51], [52]. Similarly, Goodburn [53] reports a larger clutch size related mainly to female quality with less variation explained by territory quality, and male quality explaining the variation in reproductive success. And, Sasvari and Hegyi [46] found that tawny owl (*Strix aluco*) females laid fewer eggs in snowy years, whilst older (higher quality and more experienced) females laid more eggs, and older and more experienced males provided more food, resulting in more nestlings and fledglings. In our case, in agreement with Krüger and Lindström [9], there does not seem to be an effect of experience on breeding success, although senescence could play a role not considered in our analysis (see [54]). Another possible age/experience related effect is lower performance by young/immature individuals, which has been well established in several raptor species [3], [22], [55]. In our case we did not detect this effect. This could be due to the fact that, in saturated populations, immature animals that enter the breeding population are few, and of higher quality than other individuals of the same age, or because we excluded from our analyses individuals that were monitored only once, discarding some instances of young, inexperienced individuals and reducing their effect on our results.

We found little support for an effect of territory on the variation in reproductive success. This suggests that the weight of territory was low in our study area, although it did have some effect. Some territories may have more, or at least more accessible resources, thus helping breeding males to acquire the necessary food supplies or find better nesting sites. This effect can be permanent (i.e. important prey populations) or occasional (high flow of migrants), but individual quality seems to be able to compensate for it on most occasions. Several studies on raptors reported an important effect of territory/habitat on reproductive success [3], usually estimating habitat quality using demographic measures, mainly reproductive success (37% of the studies on habitat selection reviewed by Johnson [4]). However, many studies focusing on the effect of territory only consider a few habitat categories with clear differences in quality between them in terms of variables such as prey condition and availability. Performance is usually measured at the population level, not accounting for the effect of individual quality [32]. Therefore, performance is compared between habitat categories whilst ignoring individual variation within habitat categories. This could mask important variation within a group, attributable to individual traits [19]. All these observations could be reconciled: in cases of marked or extreme differences in prey availability between territories a clear territory effect could be expected, probably with important intra group individual variability in habitat categories, whilst in other cases individual quality (and the match between individual characteristics and that of territory) could explain variation in breeding success. One important practical implication of our findings is that individual quality can often cause false apparent territory quality [32], as shown by the variance in the BQI averages of different pairs in territories 2, 4, 6 and others (see supplementary material).

The decisions taken by individuals when selecting a particular site at which to settle have important consequences for their fitness [20], [32]. Therefore, another important question is how individuals select territories, if they do at all. Coherent with a despotic distribution model, Newton [3] reported changes in reproductive success associated with territories in sparrowhawks (*Accipiter nisus*) and movements from low quality territories to high quality ones by dominant individuals. Contrary to that, none of the birds we monitored switched territory, regardless of some instances of consecutive reproductive failure. Our results are similar to those reported by Krüger and Lindström [9] in an 11 year study on the common buzzard (*Buteo buteo*). Over the study period we witnessed several cases of foreign falcons displacing territorial birds and occupying their territories, although the most common outcome was the owner rejecting the intruder [28]. The average recruitment age in our study area was 3.7 calendar years for males and 4.0 cy for females [3], thus floater peregrines looking for a territory must fight against experienced territory holders. The main cliffs and other suitable nesting areas were occupied within our study site and during the 16 years of monitoring no permanent new territory was established, despite a few trials on small cliffs. This suggests a shortage of free territories in the area. Hence, contrary to what could be expected from a despotic distribution pattern, floater peregrines probably do not select a territory to settle in, but instead occupy vacancies or territories with weak owners. Similarly, territory owners would risk their territory and also their lives in trying to move to a better territory, presuming that they could somehow assess territory quality. Several non-exclusive hypotheses could explain the observed lack of mobility of birds between territories: (1) lack of differences in quality between territories or lack of individual capability to assess it; (2) a queue hypothesis in which it would be easier and more rewarding for individuals to maintain already acquired territories than to fight for new ones; (3) a possibility of improved breeding through better knowledge of the territory and its resources, resulting from experience, although the lack of statistical significance of estimated experience in the models does not support this; (4) since lifetime reproductive success is related to lifespan [2], [3] individuals may optimise their survival probabilities and wait for more favourable breeding seasons [56], [57]; and (5) an existing match between individual and territory qualities, although this seems improbable because of the constant selection of models with individual identity as the best fit and the scarce support obtained by models in which individual identity was nested in territory. In this line, Germain and Arcese [32] report an effect of habitat on the productivity of the song sparrow (*Melospiza melodia*). However, this effect was much smaller than the effect of individual quality, and not closely related to it, indicating that the ability of birds to settle at preferred breeding sites was independent of their intrinsic quality [32].

In terms of breeding success, models considering individual identity outperformed models considering territorial identity, as well as models with no random factors, indicating that empirical evidence in the data supported the hypothesis that individual quality was the most important source of variation in breeding success, and of greater importance than territory quality. If the variation in breeding success in different territories was attributable to the different traits of the individuals, this could have been an important factor favouring and maintaining intraspecific variability and population plasticity, as opposed to traditional models in which the selection process is unidirectional.

## Supporting Information

Table S1 **reproductive success of different pairs and individuals.** “Ter” indicates territories, “Breed. pairs” stands for unique breeding pair combinations within each territory, and Male and Female for different individuals within the same territory. “Aver. Fledl” indicates the average number of fledglings produced during the period that it was successfully monitored, with standard deviation in brackets. BQI stands for the average Breeding Quality Index of the pair/individual during the period that it was successfully tracked, with standard deviation in brackets, and n indicates the number of years for which the pair or individual was monitored during the study period.
(DOC)

## References

[1] McGraw JB, Caswell H (1996) Estimation of individual fitness from life-history data. American Naturalist 147: 47–64.

[2] Clutton-Brock TH (1988) Reproductive success. Studies of individual variation in contrasting breeding systems; Clutton-Brock TH, editor. Chicago: The University of Chicago Press.

[3] Newton I (1989) Lifetime reproduction in birds. London: Academic Press.

[4] Johnson MD (2007) Measuring habitat quality: A review. Condor 109: 489–504.

[5] Martin TE (1998) Are microhabitat preferences of coexisting species under selection and adaptive? Ecology 79: 656–670.

[6] Pulliam HR (2000) On the relationship between niche and distribution. Ecology Letters 3: 349–361.

[7] Wiens JA (1989) The ecology of bird communities.Vol. I. Foundations and patterns. Cambridge: Cambridge University Press.

[8] Jones J (2001) Habitat selection studies in avian ecology: A critical review. Auk 118: 557–562.

[9] Krüger O, Lindström J (2001) Lifetime reproductive success in common buzzard, *Buteo buteo*: from individual variation to population demography. Oikos 93: 260–273.

[10] Sergio F, Newton I (2003) Occupancy as a measure of territory quality. Journal of Animal Ecology 72: 857–865.

[11] Hogstedt G (1980) Evolution of clutch size in birds - Adaptative variation in relation to territory quality. Science 210: 1148–1150. 17831471 10.1126/science.210.4474.1148

[12] Ferrer M, Donázar JA (1996) Density-dependent fecundity by habitat heterogeneity in an increasing population of Spanish Imperial Eagles. Ecology 77: 69–74.

[13] Krüger O, Chakarov N, Nielsen JT, Looft V, Grunkorn T, et al (2012) Population regulation by habitat heterogeneity or individual adjustment? Journal of Animal Ecology 81: 330–340. 21950339 10.1111/j.1365-2656.2011.01904.x

[14] Carrete M, Sánchez-Zapata JA, Tella JL, Gil-Sánchez JM, Moleón M (2006) Components of breeding performance in two competing species: habitat heterogeneity, individual quality and density-dependence. Oikos 112: 680–690.

[15] Newton I (1991) Habitat variation and population regulation in sparrowhawks. Ibis 133: 76–88.

[16] Pagán I, Martínez JE, Calvo JF (2009) Territorial occupancy and breeding performance in a migratory raptor do not follow ideal despotic distribution patterns. Journal of Zoology 279: 36–43.

[17] Espie RHM, James PC, Oliphant LW, Warkentin IG, Lieske DJ (2004) Influence of nest-site and individual quality on breeding performance in Merlins *Falco columbarius* . Ibis 146: 623–631.

[18] Bolnick DI, Svanback R, Fordyce JA, Yang LH, Davis JM, et al (2003) The ecology of individuals: Incidence and implications of individual specialization. American Naturalist 161: 1–28. 10.1086/343878 12650459

[19] Cam E, Link WA, Cooch EG, Monnat JY, Danchin E (2002) Individual covariation in life-history traits: Seeing the trees despite the forest. American Naturalist 159: 96–105. 10.1086/324126 18707403

[20] Cardador L, Carrete M, Mañosa S (2012) Inter-Individual Variability and Conspecific Densities: Consequences for Population Regulation and Range Expansion. Plos One 7: e33375 doi:10.1371/journal.pone.0033375 22479391 10.1371/journal.pone.0033375 PMC3314022

[21] Carrete M, Tella JL, Sánchez-Zapata JA, Moleón M, Gil-Sánchez JM (2008) Current caveats and further directions in the analysis of density-dependent population regulation. Oikos 117: 1115–1119.

[22] Margalida A, Mañosa S, González LM, Ortega E, Sánchez R, et al (2008) Breeding of non-adults and effects of age on productivity in the Spanish Imperial Eagle *Aquila adalberti* . Ardea 96: 173–180.

[23] Nussey DH, Coulson T, Festa-Bianchet M, Gaillard JM (2008) Measuring senescence in wild animal populations: towards a longitudinal approach. Functional Ecology 22: 393–406.

[24] Margalida A, Bertran J, Heredia R (2009) Diet and food preferences of the endangered Bearded Vulture *Gypaetus barbatus*: a basis for their conservation. Ibis 151: 235–243.

[25] Margalida A, Benítez JR, Sánchez-Zapata JA, Ávila E, Arenas R, et al (2012) Long-term relationship between diet breadth and breeding success in a declining population of Egyptian Vultures *Neophron percnopterus* . Ibis 154: 184–188.

[26] Newton I (2013) Bird Populations. London: The New Naturalist Library.

[27] Zuberogoitia I, Martínez JA, Azkona A, Martínez JE, Castillo I, et al (2009) Using recruitment age, territorial fidelity and dispersal as decisive tools in the conservation and management of peregrine falcon (*Falco peregrinus*) populations: the case of a healthy population in Northern Spain. Journal of Ornithology 150: 95–101.

[28] Zuberogoitia I, Ruiz Moneo F, Torres JJ (2002) El Halcón Peregrino. Bilbao: Servicio Publicaciones de la Diputación Foral de Bizkaia.

[29] Zuberogoitia I, Martínez JE, Zabala J (2014) Individual recognition of territorial peregrine falcons *Falco peregrinus*: a key for long-term monitoring programmes. Munibe 61: 117–127.

[30] Zuberogoitia I, Martínez JE, González-Oreja JA, Calvo JF, Zabala J (2013) The relationship between brood size and prey selection in a Peregrine Falcon population located in a strategic region on the Western European Flyway. Journal of Ornithology 154: 73–82.

[31] Martínez JE, Martínez JA, Zuberogoitia I, Zabala J, Redpath SM, et al (2008) The effect of intra- and interspecific interactions on the large-scale distribution of cliff-nesting raptors. Ornis Fennica 85: 13–21.

[32] Germain RR, Arcese P (2014) Distinguishing individual quality from habitat preference and quality in a territorial passerine. Ecology In press. 10.1890/13-0467.1 24669736

[33] Forslund P, Part T (1995) Age and reproduction in birds - Hypotheses and tests. Trends in Ecology & Evolution 10: 374–378. 21237076 10.1016/s0169-5347(00)89141-7

[34] Forsman D (1999) The raptors of Europe and the Middle East. London: T &AD Poyser.

[35] Cam E, Monnat JY (2000) Apparent inferiority of first-time breeders in the kittiwake: the role of heterogeneity among age classes. Journal of Animal Ecology 69: 380–394.

[36] Zuur AF, Ieno EN, Walker NJ, Saveliev AA, Smith GM (2009) Mixed effects models and extensions in ecology with R.; Gail M, Krickeberg K, Samet JM, Tsiatis A, Wong W, editors. New York: Springer.

[37] Bolker BM, Brooks ME, Clark CJ, Geange SW, Poulsen JR, et al (2009) Generalized linear mixed models: a practical guide for ecology and evolution. Trends in Ecology & Evolution 24: 127–135. 19185386 10.1016/j.tree.2008.10.008

[38] Burnham KP, Anderson DR (2002) Model selection and multimodel inference. A practical information-theoretic approach. New York: Springer.

[39] Zar JH (1999) Biostatistcal analysis. New Jersey: Prentice Hall.

[40] Crawley MJ (2007) The R book. Chichester: Wiley.

[41] R Core Team (2013) R: A language and environment for statistical computing. R Foundation for Statistical Computing, Vienna, Austria. URL http://www.R-project.org/.

[42] Pinheiro J, Bates D, DebRoy S, Sarkar DR, Team DC (2012) lme: Linear and Nonlinear Mixed Effects Models. R package version 3.1-104 ed.

[43] Bates D, Maechler M, Bolker B (2012) lme4: Linear mixed-effects models using S4 classes. R package version 0.999999-0. ed.

[44] Olsen J, Tucker AD (2003) A brood-size manipulation experiment with Peregrine Falcons, *Falco peregrinus*, near Canberra. Emu 103: 127–132.

[45] Sasvari L, Hegyi Z, Csorgo T, Hahn I (2000) Age-dependent diet change, parental care and reproductive cost in tawny owls *Strix aluco* . Acta Oecologica 21: 267–275.

[46] Sasvari L, Hegyi Z (2002) Effects of age composition of pairs and weather condition on the breeding performance of tawny owls, *Strix aluco* . Folia Zoologica 51: 113–120.

[47] Katzner TE, Bragin EA, Knick ST, Smith AT (2005) Relationship between demographics and diet specificity of Imperial Eagles *Aquila heliaca* in Kazakhstan. Ibis 147: 576–586.

[48] Rodríguez C, Bustamante J (2003) The effect of weather on lesser kestrel breeding success: can climate change explain historical population declines? Journal of Animal Ecology 72: 793–810.

[49] Zuberogoitia I, Zabala J, Martíinez JA, Martínez JE, Azkona A (2008) Effect of human activities on Egyptian vulture breeding success. Animal Conservation 11: 313–320.

[50] Korpimaki E (1988) Costs of reproduction and success of manipulated broods under varying food conditions in Tengmalms owl. Journal of Animal Ecology 57: 1027–1039.

[51] Steenhof K, Kochert MN, McDonald TL (1997) Interactive effects of prey and weather on golden eagle reproduction. Journal of Animal Ecology 66: 350–362.

[52] Margalida A, Colomer MA, Oro D (2014) Man-induced activities modifies demographic parameters in a long-lived species: effects of poisoning and health policies. Ecological Applications In press. 10.1890/13-0414.1 24834731

[53] Goodburn SF (1991) Territory quality or bird quality - factors determining breeding success in the magpie *Pica-pica* . Ibis 133: 85–90.

[54] Millon A, Petty S, Little B, Lambin X (2011) Natal conditions alter age-specific reproduction but not survival or senescence in a long-lived bird of prey. Journal of Animal Ecology 80: 968–975. 21466554 10.1111/j.1365-2656.2011.01842.x

[55] Ferrer M, Bisson I (2003) Age and territory-quality effects on fecundity in the Spanish Imperial Eagle (*Aquila adalberti*). Auk 120: 180–186.

[56] Zabala J, Zuberogoitia I, Martínez-Climent JA, Etxezarreta J (2011) Do long lived seabirds reduce the negative effects of acute pollution on adult survival by skipping breeding? A study with European storm petrels (*Hydrobates pelagicus*) during the "Prestige" oil-spill. Marine Pollution Bulletin 62: 109–115. 20884034 10.1016/j.marpolbul.2010.09.004

[57] Crone EE (2001) Is survivorship a better fitness surrogate than fecundity? Evolution 55: 2611–2614. 11831674 10.1111/j.0014-3820.2001.tb00773.x

