# Supplementary material for: Individual Quality Explains Variation in Reproductive Success Better than Territory Quality in a Long-Lived Territorial Raptor
Source: PLoS One. 2014 Mar 5;9(3):e90254. doi: 10.1371/journal.pone.0090254 (PMC3943913; doi:10.1371/journal.pone.0090254)
Supplement: Table S1 — reproductive success of different pairs and individuals. “Ter” indicates territories, “Breed. pairs” stands for unique breeding pair combinations within each territory, and Male and Female for different individuals within the same territory. “Aver. Fledl” indicates the average number of fledglings produced during the period that it was successfully monitored, with standard deviation in brackets. BQI stands for the average Breeding Quality Index of the pair/individual during the period that it was successfully tracked, with standard deviation in brackets, and n indicates the number of years for which the pair or individual was monitored during the study period. (DOC) [file pone.0090254.s001.doc]

Table S1: reproductive success of different pairs and individuals. “Ter” indicates territories, “Breed. pairs” stands for unique breeding pair combinations within each territory, and Male and Female for different individuals within the same territory. “Aver. Fledl” indicates the average number of fledglings produced during the period that it was successfully monitored, with standard deviation in brackets. BQI stands for the average Breeding Quality Index of the pair/individual during the period that it was successfully tracked, with standard deviation in brackets, and n indicates the number of years for which the pair or individual was monitored during the study period.

| **Ter** | **Breed. pair** | **Aver. nestl.** | **BQI** | **n** | **Male** | **Aver. Fledl.** | **BQI** | **n** | **Fem.** | **Aver. Fledl.** | **BQI** | **n** |
| --- | --- | --- | --- | --- | --- | --- | --- | --- | --- | --- | --- | --- |
| 1 | 1 | 2.7 (1.16) | 0.61 (0.87) | 10 | 1 | 2.7 (1.15) | 0.60 (1.01) | 10 | 1 | 2.63 (1.12) | 0.60 (0.82) | 11 |
| 1 |  |  |  |  | 2 | 0.5 (1) | -0.8 (1.10) | 4 |  |  |  |  |
| 2 | 2 | 3.67 (0.58) | 0.95 (0.65) | 3 | 3 | 3.67 (0.58) | 0.95 (0.65) | 3 | 2 | 2.8 (1.64) | 0.23 (1.6) | 5 |
| 2 | 3 | 1.5 (2.12) | -0.85 (2.34) | 2 | 4 | 0.75 (1.5) | -1.49 (1.59) | 4 | 3 | 0.5 (1) | -1.52 (0.97) | 4 |
| 2 | 4 | 0 (0) | -2.13 (0.65) | 2 |  |  |  |  |  |  |  |  |
| 3 | 5 | 2.75 (1.49) | 1.19 (1.24) | 8 | 5 | 2.75 (1.49) | 1.19 (1.24) | 8 | 4 | 2.75 (1.49) | 1.19 (1.24) | 8 |
| 4 | 6 | 1.5 (0.71) | -0.06 (0.91) | 2 | 6 | 1 (1) | -0.6 (1.13) | 3 | 5 | 1.5 (0.71) | -0.06 (0.91) | 2 |
| 4 | 7 | 2.33 (2.08) | 1.04 (2.33) | 3 | 7 | 2.33 (2.08) | 1.04 (2.33) | 3 | 6 | 2.33 (2.08) | 1.04 (2.33) | 3 |
| 5 | 8 | 2.33 (0.58) | 1.04 (0.65) | 3 | 8 | 2.33 (0.58) | 1.04 (0.65) | 3 | 7 | 2.33 (0.58) | 1.04 (0.65) | 3 |
| 6 | 9 | 2.4 (1.34) | 0.27 (0.37) | 5 | 9 | 2.4 (1.34) | 0.26 (1.13) | 5 | 8 | 3 (0) | 0.34 (0.39) | 5 |
| 6 | 10 | 0 (0) | -1.4 (0.44) | 4 | 10 | 1 (1.41) | -1.3 (0.34) | 6 | 9 | 0 (0) | -1.4 (0.44) | 4 |
| 6 |  |  |  |  | 11 | 0 (0) | -1.4 (0.96) | 2 |  |  |  |  |
| 7 | 11 | 2.67 (1.03) | 0.54 (0.93) | 6 | 12 | 2.67 (1.03) | 0.54 (0.92) | 6 | 10 | 2.18 (1.08) | 0.38 (0.76) | 11 |
| 7 | 12 | 1.5 (1) | 0.1 (0.6) | 4 | 13 | 1.5 (1) | 0.1 (0.6) | 4 |  |  |  |  |
| 8 | 13 | 2.33 (1.53) | 0.18 (1.09) | 3 | 14 | 2.33 (1.53) | 0.17 (1.1) | 3 | 11 | 2.33 (1.53) | 0.17 (1.1) | 3 |
| 8 |  |  |  |  | 15 | 2.5 (0.70) | 0.93 (0.45) | 2 | 12 | 2.33 (1.15) | 0.73 (1.24) | 3 |
| 8 |  |  |  |  | 16 | 3 (0) | 1.90 (1.03) | 2 |  |  |  |  |
| 9 | 14 | 1 (1.73) | -0.58 (1.87) | 3 | 17 | 1 (1.26) | -0.44 (1.29) | 6 | 13 | 1.25 (1.5) | -0.49 (1.54) | 4 |
| 9 | 15 | 1 (1) | -0.3 (0.76) | 3 |  |  |  |  | 14 | 1 (1) | -0.3 (0.76) | 3 |
| 10 | 16 | 1.5 (2.12) | -0.06 (1.91) | 2 | 18 | 1.75 (1.5) | -0.27 (1.58) | 4 | 15 | 1.5 (2.12) | -0.06 (1.91) | 2 |
| 10 | 17 | 0 (0) | -1.56 (0.19) | 2 | 19 | 1.5 (2.12) | 0.0 (0.46) | 2 | 16 | 0 (0) | -1.56 (0.19) | 2 |
| 10 |  |  |  |  | 20 | 0 (0) | -1.3 (0.47) | 3 |  |  |  |  |
| 11 | 18 | 2.5 (0.71) | 0.99 (0.57) | 2 | 21 | 1.67 (1.53) | 0.09 (1.61) | 3 | 17 | 2.5 (0.71) | 0.99 (0.57) | 2 |
| 12 | 19 | 1 (1.73) | -0.07 (0.13) | 3 | 22 | 2.38 (1.3) | -0.04 (1.3) | 10 | 18 | 2.1 (1.37) | 0.02 (1.37) | 10 |
| 12 | 20 | 2.29 (1.38) | -0.01 (1.4) | 7 | 23 | 1.67 (1.53) | 0.06 (1.65) | 3 | 19 | 1 (1.73) | -0.07 (0.13) | 3 |
| 12 | 21 | 2.5 (0.71) | 0.94 (0.91) | 2 |  |  |  |  | 20 | 0 (0) | -1.0 (0.45) | 2 |
| 13 | 22 | 0.75 (0.96) | -0.81 (1.02) | 4 | 24 | 0.75 (0.96) | -0.81 (1.02) | 4 | 21 | 0.75 (0.96) | -0.81 (1.02) | 4 |
| 14 | 22 | 0.5 (0.71) | -1.01 (0.84) | 2 | 25 | 1 (1.41) | -0.95 (1.17) | 4 | 22 | 0.33 (0.58) | -1.25 (0.72) | 3 |
| 15 | 23 | 3 (1.41) | 0.7 (1.43) | 7 | 26 | 3 (1.41) | 0.7 (1.44) | 7 | 23 | 2.46 (1.51) | 0.56 (1.35) | 13 |
| 15 | 24 | 2.5 (0.71) | 0.99 (0.57) | 2 | 27 | 2.5 (0.70) | 0.98 (2.13) | 2 |  |  |  |  |
| 15 | 25 | 1.5 (2.12) | -0.2 (2.14) | 2 | 28 | 1.5 (2.12) | -0.2 (1.66) | 2 |  |  |  |  |
| 15 | 26 | 1.5 (2.12) | 0.4 (1.67) | 2 | 29 | 1.5 (2.12) | 0.40 (1.34) | 2 |  |  |  |  |
| 16 | 27 | 2 (1.73) | 0.39 (1.57) | 3 | 30 | 2.25 (1.5) | 0.64 (1.37) | 4 | 24 | 2.2 (1.3) | 0.8 (1.24) | 5 |
| 17 | 28 | 3 (1.41) | 1.07 (1.04) | 2 | 31 | 3 (1.41) | 1.07 (1.04) | 2 | 25 | 3 (1.41) | 1.07 (1.04) | 2 |
| 17 | 29 | 2 (1.41) | 0.39 (1.3) | 4 | 32 | 2.2 (1.3) | 0.63 (1.25) | 5 | 26 | 2 (1.41) | 0.39 (1.3) | 4 |
| 18 | 30 | 1.33 (1.53) | -0.49 (1.59) | 3 | 33 | 1.33 (1.53) | -0.5 (1.6) | 3 | 27 | 2 (1.83) | -0.02 (1.62) | 4 |
| 19 |  |  |  |  | 34 | 2 (2.82) | -0.1 (2.17) | 2 |  |  |  |  |
| 20 |  |  |  |  | 35 | 1 (1.73) | -0.6 (1.64) | 3 | 28 | 0 (0) | -1.5 (0.19) | 2 |
| 21 |  |  |  |  | 36 | 0.75 (0.95) | -0.8 (1.05) | 4 | 29 | 2 (1.67) | -0.2 (1.48) | 6 |
| 21 |  |  |  |  |  |  |  |  | 30 | 1 (1.41) | -0.5 (1.60) | 2 |
| 22 |  |  |  |  | 37 | 2 (2.82) | 0.29 (2.84) | 2 | 31 | 2.5 (1) | 0.26 (0.64) | 4 |
| 23 |  |  |  |  |  |  |  |  | 32 | 2.57 (0.78) | 0.12 (0.88) | 7 |
| 24 |  |  |  |  |  |  |  |  | 33 | 2.83 (0.75) | 1.11 (0.80) | 6 |
| 25 |  |  |  |  |  |  |  |  | 34 | 2.25 (0.5) | 0.23 (0.58) | 4 |
| 26 |  |  |  |  |  |  |  |  | 35 | 1 (1) | -1.1 (0.54) | 3 |
| 26 |  |  |  |  |  |  |  |  | 36 | 1 (1.41) | -0.5 (1.55) | 2 |
| 27 |  |  |  |  |  |  |  |  | 37 | 2.66 (0.57) | 0.51 (0.99) | 3 |
| 28 |  |  |  |  |  |  |  |  | 38 | 1.33 (2.30) | 0.03 (1.99) | 3 |
